# Exploring the clinical complexity of cardio-facio-cutaneous syndrome: insights from a pediatric case series

**DOI:** 10.3389/fped.2024.1355277

**Published:** 2024-05-27

**Authors:** Yuexu Ou, Jie Cao, Yuanhui Duan, FengHua Chen, Jiwei Zhou, Jieling Li, Xiaoming Gan

**Affiliations:** ^1^ Department of General Medicine, Children’s Hospital of Chongqing Medical University, Chongqing, China; ^2^ China International Science and Technology Cooperation Base of Child Development and Critical Disorders, Chongqing, China; ^3^ National Clinical Research Center for Child Health and Disorders, Chongqing, China

**Keywords:** *BRAF* gene, cardio-facio-cutaneous syndrome, RASopathies, developmental delay, seizures

## Abstract

**Background:**

Cardio-Facio-Cutaneous syndrome (CFCS) is a rare autosomal dominant genetic disorder primarily caused by BRAF gene mutations, posing diagnostic challenges due to its multifaceted clinical presentation.

**Objective:**

To elucidate the clinical characteristics of pediatric CFCS patients, expanding the phenotypic spectrum to enhance early diagnostic capabilities, while also presenting the relationship between genotye and corresponding phenotype severity.

**Methods:**

From January 2015 to March 2022, four children diagnosed with CFCS in Children's Hospital of Chongqing Medical University were included for analysis. Whole exome sequencing (WES) was conducted to identify the types and locations of possible gene mutations. Neurological development was assessed using electroencephalography (EEG), magnetic resonance imaging (MRI) and Gesell developmental evaluation.

**Results:**

All four CFCS patients exhibited *de novo BRAF* gene mutations, manifesting with cardiac malformations, distinctive facial features, skin and hair changes, and neurological abnormalities. WES revealed that the specific *BRAF* mutations were closely linked to their clinical severity. Three patients displayed milder symptoms (case 1–3, genotype I or II), demonstrating stability or slight improvement, whereas one patient (case 4, genotype III) suffered from a severe phenotype characterized by profound neurological and digestive system impairments, leading to a significantly reduced quality of life and a grim prognosis.

**Conclusion:**

In CFCS patients, severe developmental delay and seizures are predominant neurological features, possibly accompanied by continuous spike-and-wave during sleep (CSWS) and severe sleep disturbances. CFCS generally carries a poor prognosis, underscoring the importance of disease awareness and early genetic testing.

## Introduction

CFCS is an uncommon autosomal dominant genetic disorder characterized by a group of core symptoms, including distinctive facial features, cardiac malformations, skin and hair abnormalities, and neurological anomalies (1). It is already known that CFCS is part of a group of syndromes caused by mutations in genes such as *BRAF, KRAS, MAP2K1*, and *MAP2K2*, leading to disruptions in the Ras/Mitogen-Activated Protein Kinase (RAS/MAPK) signaling pathway (2, 3). Among these, CFCS caused by BRAF gene mutations is the most common, accounting for 75% (4, 5). It is classified as one of the RAS pathway diseases (RASopathies). The overall prevalence of RASopathies is reported to be 1/1,000, with CFCS specifically affecting 1/810,000 individuals (6). Due to its extreme rarity and the clinical heterogeneity, along with overlaps with other RAS pathway disorders (7), CFCS is poorly understood in terms of its clinical phenotype, making diagnosis and differentiation quite challenging.

CFCS is a multisystem clinical syndrome that notably affects the nervous system extensively, leading to severe intellectual disabilities, behavioral abnormalities, and a high epilepsy incidence of up to 64%, accompanied by epileptic encephalopathy (8). Additionally, there is even a potential for the development of neoplastic disorders such as acute lymphoblastic leukemia and lymphomas (9).

**Figure 1:**
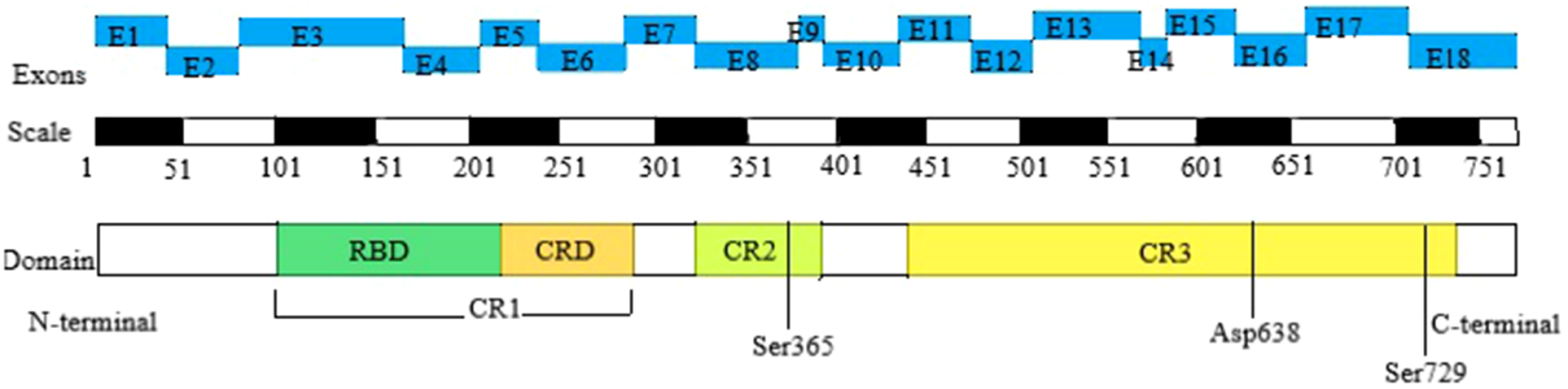
Domain organization of BRAF in CFCS. The top row depicts the positions of the 18 exons of the BRAF gene, the second row shows the corresponding amino acid numbers, and the third row presents the key structural domains of BRAF. The N-terminal CR1 domain contains the region mediating binding with activated RAS (RAS-binding domain, RBD) and the cysteine-rich domain (CRD), which serves a regulatory function. The C-terminal CR3 region encompasses the catalytic site and Ser 729, which serves as a binding site for 14-3-3 proteins when phosphorylated. The CR2 region contains a second 14-3-3 binding site (Ser 365), which plays a critical role in regulating the switch between the catalytically inactive and active conformations. CFCS, cardio-facio-cutaneous syndrome; RBD, RAS-binding domain; CRD, cysteine-rich domain; CR1, cysteine-rich 1; CR2, cysteine-rich 2; CR3, cysteine-rich 3.

The *BRAF* gene is located on human chromosome 7q34 and consists of 18 exons, encoding a protein with 651 amino acids. Currently, there are over 82 known pathogenic variants of the *BRAF* gene. The *BRAF* kinase comprises three conserved regions, namely CR1, CR2, and CR3, each representing distinct functional domains. CR1 includes the RAS-binding domain (RBD) and the cysteine-rich domain (CRD), serving as the primary regulatory functional motifs. CR2 contains the 14-3-3 protein phosphorylation binding site and regulates the transition between non-active and active conformations. CR3 houses the catalytic site and the 14-3-3 protein phosphorylation binding site. Therefore, CFCS with BRAF mutation is further divided into 3 subtypes. Type I primarily affects residues in or near the CRD, which interacts with 14-3-3 proteins (specifically Thr241, Leu245, Ala246, Gln257, Asp565, Gln709). Type II disrupts the autoinhibitory conformation of BRAF, impacting residues within the inhibitory region (Phe468, Gly469, Thr470, Lys483, Leu485, Val487, Lys499, Glu501, Leu525, Trp531, Asn581, Phe595, Thr599, Lys601). As for Asp638, it is independently located and classified as Type III (8, 10) (Figure 1).

Existing research suggests a notable correlation between the severity of clinical phenotypes in CFCS and its specific subtype, with Type I potentially presenting as a milder form and Type II and III likely manifesting as more severe forms (8). Consequently, CFCS is characterized by significant genetic and phenotypic heterogeneity, necessitating further investigations into the relationship between genotype and clinical phenotype.

In this study, we analyzed a case series of 4 children with CFCS attributed to *BRAF* gene mutations. We also summarized and analyzed their clinical characteristics, discovering some new clinical features that broaden the phenotypic spectrum of CFCS. This may enhance the awareness of clinical physicians and suggest a certain correlation between the severity of CFCS clinical phenotypes and specific gene location.

## Materials and methods

Ethical approval was obtained from the Children's Hospital of Chongqing Medical University Ethics Committee, and informed consent was obtained from the patients' guardians.

### Inclusion and exclusion criteria

Between January 2015 and March 2022, patients presenting with symptoms of cardiac malformations, distinctive facial features, skin and hair changes, and neurological abnormalities were reviewed from our electronical database. Patients diagnosed with CFCS by gene test were included for further analysis. Exclusion criteria include: patients not underwent gene testing, noonan Syndrome (NS), noonan syndrome with multiple lentigines (NSML), neurofibromatosis type I (NF1), costello syndrome (CS) and Legius syndrome (LS).

#### Genetic testing method

Peripheral venous blood samples (2 ml)were collected from each patient and their parents using EDTA anticoagulant tubes for subsequent whole exome sequencing. WES was conducted using high-throughput sequencing technology with the IDT The xGen Exome Research Panel v1.0 exome capture chip, and sequencing was performed on the Illumina NovaSeq 6,000 series sequencer. The target sequence coverage was maintained at a minimum of 99%. Subsequently, bioinformatics and clinical information analysis techniques were employed using the comprehensive spectrum of precision diagnosis cloud platform system to analyze and screen the generated data. This involved the application of genetic data analysis algorithms derived from pathogenic mutation databases, normal human genome databases, and databases containing known clinical features of thousands of genetic diseases. Variants were classified utilizing a three-tier classification system and the American college of medical genetics (ACMG) gene variant classification system. Suspected pathogenic mutations were further validated using Sanger sequencing. The target sequences were PCR-amplified and subjected to Sanger sequencing utilizing the ABI3730 sequencer, with the results obtained through sequence analysis software.

#### Clinical data

All patients underwent comprehensive routine tests and Gesell developmental evaluation, aimed at understanding their medical condition and excluding other potential underlying causes. Extensive clinical data were gathered for all four cases, encompassing age, gender, birth history, growth and developmental history, clinical manifestations, imaging data, laboratory test results, and genetic testing outcomes. Subsequently, the patients' treatment and prognosis were monitored and followed up.

#### Mutation analysis

Regarding the variant positions identified in the sequencing results, the nature of these mutations is determined by cross-referencing various databases, including the Online Mendelian Inheritance in Man (OMIM), the Single Nucleotide Polymorphism Database (dbSNP), the Human Gene Mutation Database (HGMD), and the NSEuroNet database. This comprehensive analysis is complemented by an assessment of clinical presentations, aiming to explore the intricate connection between genotypes and clinical phenotypes.

## Results

### Clinical presentations and genetic testing results

The demographic features and clinical manifestations were presented in Table 1 and Supplementry Figure S1. None of their parents had consanguineous marriages, and there were no significant maternal pregnancy histories or family history of the disease. Case 4 patient had severe malnutrition due to feeding difficulties, with a weight less than −3 SD and height at the median. Regarding epilepsy, Case 4 patient had intractable seizures, while Case 2 patient had afebrile seizures. Although Case 1 and 3 did not experience seizures, they exhibited severe sleep disturbances, characterized by bedtime excitement, difficulty falling asleep, reduced sleep duration, nighttime crying, etc. Their electroencephalograms (EEGs) showed continuous spike-and-wave during sleep (CSWS) patterns. Brain MRI revealed cerebral atrophy, significant enlargement of the periventricular white matter, and thinning of the corpus callosum in some cases. Among the four cases, three presented with cardiac malformations, primarily characterized by small atrial septal defects.

**Table 1:** Clinical Presentation of 4 Cases of CFC Patients.

Patient ID	Case 1	Case 2	Case 3	Case 4
Age at diagnosis	1 year 2 months	4 months	2 years	1 year 3 months
Gender	Male	Male	Female	Female
BRAF mutation site	ch7:140477806, exon12, c.1502A > G (p.Glu501Gly)	ch7:140453133, exon15, c.1802A > T (p.Lys601Ile)	ch7:140453133, exon6, c.722C > T (p.Thr241Met)	chr7:140501350, exon16, c.1914T > G (p.Asp638Glu)
Genotype	Type II	Type II	Type I	Type III
Cardiac abnormalities	ASD, ventricular septum and left ventricular wall thicken	ASD, Pulmonary artery stenosis	−	ASD
Distinctive facial features	+	+	+	+
Skin and hair abnormalities	+	+	+	+
Developmental delay	+	+	+	+
Gesess (DQ score)				
Gross motor	50	50	31	11
Fine motor	36	58	37	0
Reaction to objects	30	58	32	5
Reaction to people	33	42	30	3
Language	31	33	30	2
Epilepsy	−	+	−	+
Abnormal EEG	+	+	+	+
Head MRI	Delayed myelination	Thinning of the corpus callosum	Thinning of the corpus callosum; Brain atrophy.	Paraventricular white matter dysplasia; Brain atrophy.
Other system abnormalities	Subclinical hypothyroidism; laryngeal achondroplasia; sleep disturbances; intermittent exotropia; nystagmus	Cryptorchidism; short stature	Sleep disturbances	Feeding difficulty; severe malnutrition

“+” indicates the presence of a condition, “−“ indicates its absence.

AEDs, anti-epileptic drugs; ASD, atrial septal defect; EEG, electroencephalograms.

WES revealed that all four patients had *de novo* mutations in the *BRAF* gene located on chromosome 7. The pathogenicity of these mutations was suggested by ACMG classification. Although the parents of the patients had wild-type alleles, the specific mutation sites varied among them. The *BRAF* gene mutation sites in the four patients were described in Table 1.

### Treatment and follow-up status

Since all four patients exhibited the core symptoms of “heart-facial-skin-hair-neurological” manifestations and were found to have *BRAF* gene mutations, they were eventually diagnosed with CFCS.

Notably, Case 4 had refractory epilepsy and underwent multiple treatments, yet control remained elusive. Case 2 showed improved seizure control with levetiracetam, but EEG findings remained unchanged. Additionally, Cases 1 and 3 suffered from severe sleep disturbances and exhibited EEG patterns indicative of CSWS. Treatment partially improved sleep disturbances in Case 3 but had no significant effect on EEG patterns. Case 1 did not receive antiepileptic medication, and both sleep disturbances and EEG patterns showed no improvement. Additionally, during the follow-up, we observed three cases with cardiac malformations, where the defects gradually self-resolved as the patients aged.

During the follow-up, Case 1–3 showed signs of remission. However, despite various treatment attempts, Case 4's seizures were not completely controlled, resulting in long-term bedridden status and severe malnutrition, significantly affecting the quality of life. Notably, the genetic mutation sites of the four cases were as follows: Case 1 harbored c.1502A > G (p.Glu501Gly), classified as type II; Case 2 had c.1802A > T (p.Lys601Ile), also classified as type II; Case 3 presented with c.722C > T (p.Thr241Met), categorized as type I; and Case 4 carried c.1914T > G (p.Asp638Glu), classified as type III.

## Discussion

Our analysis confirmed the correlation between CFCS genotypes and clinical phenotypes, highlighting the impact of specific BRAF mutation sites on disease severity. It also uncovered new neurological findings, like sleep disturbances and CSWS patterns, broadening the CFCS phenotype spectrum. Additionally, antiepileptic drugs alleviated sleep issues but didn't affect EEG CSWS patterns. Moreover, cardiac malformations in CFCS patients showed spontaneous improvement with age.

**Figure 2:**
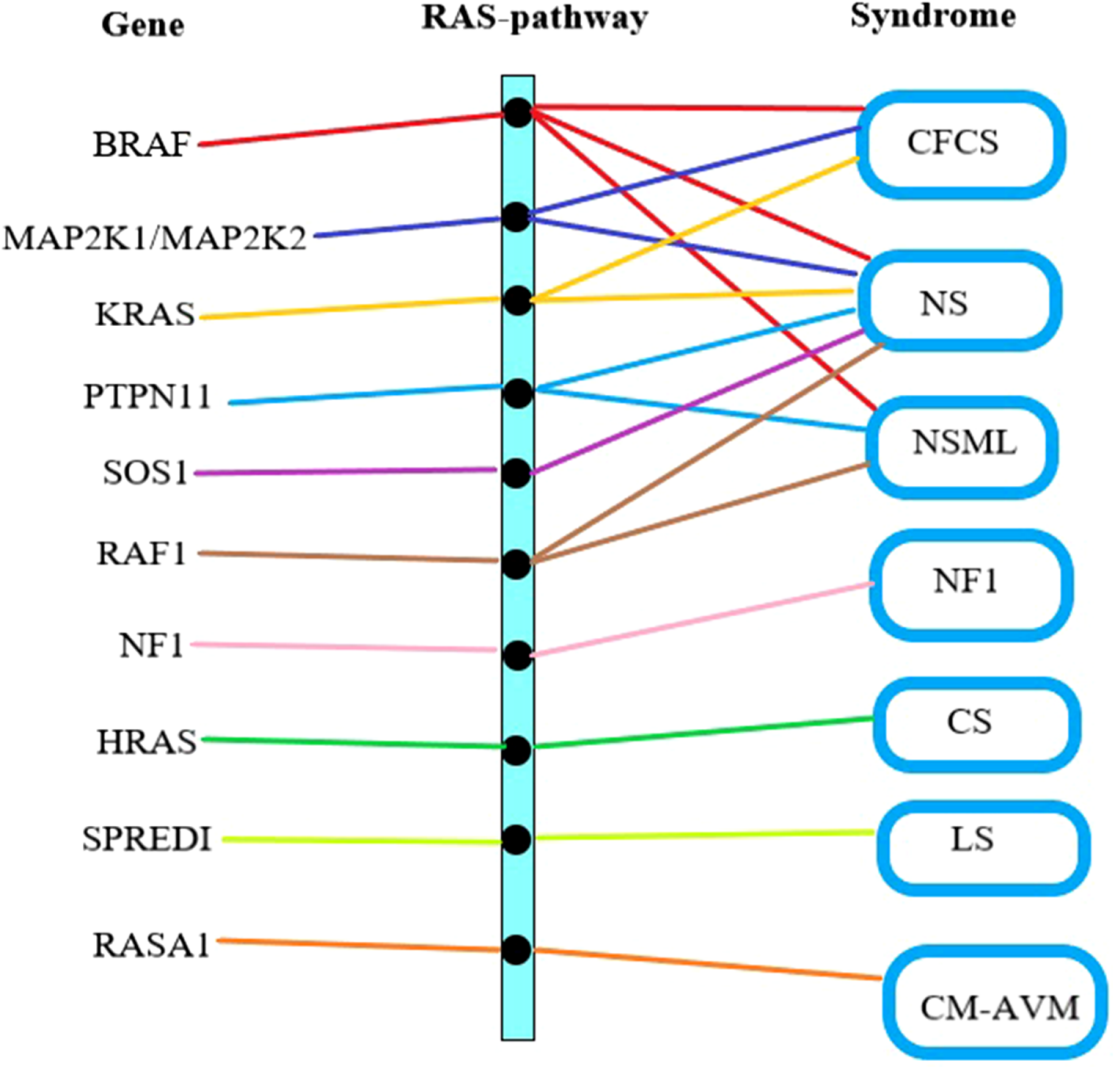
Gene mutation-RAS pathway abnormality-clinical syndrome relationship. The first column lists the gene names causing RAS pathway abnormalities, the middle column represents RAS pathway abnormalities, and the last column lists the clinical syndromes associated with RAS pathway abnormalities. CFCS, cardio-facio-cutaneous syndrome; NS, noonan syndrome; NSML, noonan syndrome with multiple lentigines; NF1, neurofibromatosis type I; CS, costello syndrome; LS, legius syndrome; CM-AVM, capillary malformation–arteriovenous malformation syndrome.

The Ras/Mitogen-Activated Protein Kinase (RAS/MAPK) signaling pathway has been proven to influences cell proliferation, differentiation, senescence, and apoptosis, which plays a crucial role in the mechanism of CFCS (11). There is a complex interplay between genes, the Ras pathway, and clinical syndromes (Figure 2). Specifically, *BRAF* gene mutations contribute to three types of disorders: CFCS, NS, and NSML (12). Genes such as *MAP2K1, MAP2K2, KRAS, PTPN11, SOS1, RAF1, NF1, HRAS, SPREDI, RASA1*, among others, can also lead to RAS pathway disorders (13). The clinical classification of RAS pathway disorders includes seven subtypes: CFCS, NS, NSML, NF1, CS, LS, and CM-AVM (14). Due to their shared pathogenic mechanism, there is partial overlap in the clinical presentation. Hence, distinguishing poses a certain challenge (15).

The four reported cases exhibited the core symptoms of CFCS with only one case not showing any cardiac anomalies. Interestingly, with increasing age, these cardiac abnormalities self-resolved, which contrasts with the literature report of CFCS patients showing pulmonary valve stenosis and hypertrophic cardiomyopathy as the most common cardiac anomalies, in which the corresponding follow-up results were not mentioned (4). The neurological abnormalities were the most prominent features in this cohort and literature reports (15, 16), with 90% displaying intellectual disabilities, and over 50% experiencing seizures and encephalopathy (4, 8). However, our study unveiled previously unreported neurological changes in CFCS, including sleep disturbances and CSWS manifestations. Nonetheless, it's crucial to acknowledge the remarkable heterogeneity of CFCS. Variations in clinical severity and associated symptoms can be attributed to diverse BRAF gene mutation sites (8, 17, 18).

The literature reports three CFCS cases with identical gene mutations to our study [Supplementary Table S1 (case 5–7)]. An Asp638 mutation case is notable for its severe CFCS phenotype, while Glu501 is characteristic of classic CFCS (19, 20). However, a Thr241 case diverges due to severe neurological symptoms (21). This contrast requires scrutiny for potential secondary cerebral damage. Two cases with Phe595Leu (type II) and Gln257Arg (type I) mutations exhibit severe CFCS phenotypes (22) [Supplementary Table S1 (case 8–9)]. Overall, while cardiac abnormalities aren't mandatory, neurological and skin/hair abnormalities are crucial in CFCS, with mutations influencing clinical severity.

Based on the clinical presentations of the four cases, differential diagnosis with NS should be considered. NS and CFCS share common pathogenic mechanisms, and their clinical manifestations overlap in many aspects. Both disorders can exhibit similar core symptoms. However, there are some distinguishing features. NS is often associated with cryptorchidism and short stature, earning it the nickname “Turner syndrome in males.” Additionally, hematological abnormalities are more common in NS. Moreover, over 50% of NS patients have *PTPN11* gene mutations (23). On the other hand, CFCS mainly manifests as abnormalities in the ectodermal tissues, such as characteristic skin and hair changes, and neurological abnormalities. In CFCS patients, approximately 75% have *BRAF* gene mutations. In the four cases described in this study, all had *BRAF* mutations, and their most prominent manifestations were related to neurological abnormalities, accompanied by characteristic skin and hair changes, aligning more closely with the diagnosis of CFCS syndrome.

One limitation of the study is the small sample size, as only four cases were included. Due to the rarity of CFCS, large-scale studies with more cases are challenging to conduct. Nevertheless, the study provides valuable insights into the clinical characteristics of CFCS caused by *BRAF* gene mutations.

In summary, CFCS linked to *BRAF* gene mutations is a rare and complex genetic disorder with diverse clinical symptoms. This study highlights the importance of recognizing core symptoms, particularly neurological abnormalties, for accurate CFCS diagnosis and management. Our analysis reaffirms the correlation between CFCS genotypes and clinical severity, driven by specific BRAF mutation sites. Additionally, we report the presence of sleep disturbances and CSWS in CFCS, as well as favorable prognoses in cases of cardiac malformations, thereby expanding its clinical spectrum. Further research and genetic studies are needed to elucidate the underlying molecular mechanisms and genotype-phenotype correlations to develop targeted therapies for these rare genetic disorders.

## Data Availability

The datasets presented in this article are not readily available because of patient privacy. Requests to access the datasets should be directed to the corresponding author.
