# Supplementary figures and images for: Exploring the clinical complexity of cardio-facio-cutaneous syndrome: insights from a pediatric case series
Source: Front Pediatr. 2024 May 27;12:1355277. doi: 10.3389/fped.2024.1355277 (PMC11163133; doi:10.3389/fped.2024.1355277)

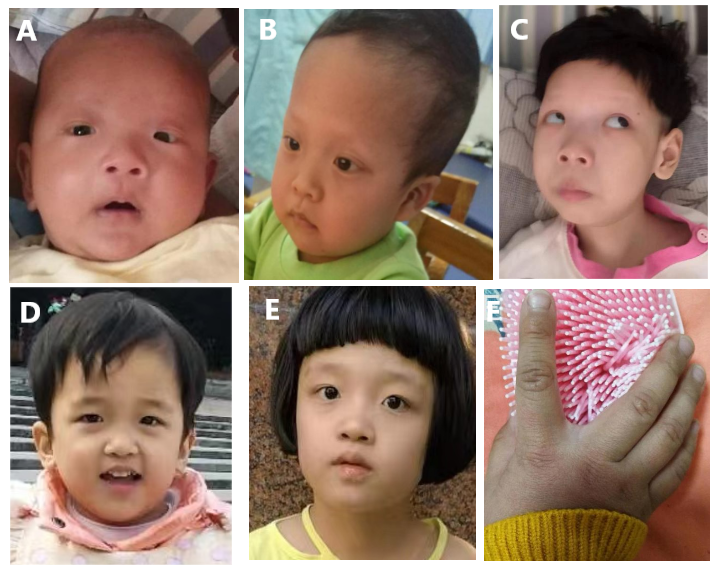

Supplement: Supplementary file 1 [file Image1.tiff]
